# Treatment of a young child with multicentric carpotarsal osteolysis exhibiting joint inflammation and dysfunctional bone formation

**DOI:** 10.1016/j.bonr.2023.101701

**Published:** 2023-07-19

**Authors:** Bailey Trinkino, Nina S. Ma

**Affiliations:** a Marian University College of Osteopathic Medicine, 3200 Coldspring Road, Indianapolis, IN 46222, United States of America; b Section of Endocrinology, Children's Hospital Colorado, 13123 E. 16th Avenue, Aurora, CO 80045, United States of America; c Department of Pediatrics, University of Colorado School of Medicine, 13001 E. 17th Place, Aurora, CO 80045, United States of America

**Keywords:** MCTO, MAFB, Osteolysis, Inflammation, Bone formation

## Abstract

Multicentric carpotarsal osteolysis (MCTO) is a rare skeletal dysplasia characterized by osteolysis of the carpal and tarsal bones. Antiresorptive agents have proven ineffective and the pathogenesis of MCTO remains poorly understood. We report a young child with a novel variant in *MAFB* who demonstrated clinical improvement of joint symptoms following anti-rheumatic therapies. Also, radiographs from a young age suggest that dysfunctional bone formation may play a role in the skeletal phenotype of MCTO.

## 1 Introduction

Multicentric carpotarsal osteolysis (MCTO; OMIM #166300) is an ultra-rare skeletal dysplasia caused by heterozygous missense mutations within a narrow region of the transactivation domain of *MAFB* (v-maf musculoaponeurotic fibrosarcoma oncogene ortholog B) (Zankl et al., 2012). MafB is a member of the large Maf family of basic leucine zipper transcription factors that contain a transcriptional activation domain, basic DNA binding domain, and leucine zipper structure for dimer formation. Maf proteins form homo- and heterodimers that bind to DNA sequences called Maf recognition elements and regulate target gene transcription (Takahashi, 2021).

MCTO is clinically characterized by the progressive destruction of carpal and tarsal bones, renal disease with a high rate of renal failure (Wu et al., 2021), and subtle craniofacial differences, such as triangular face, micrognathia, thin upper lip, hypoplastic nares, and exophthalmos. The bone disease in MCTO begins in early childhood with a median age of onset of two years (Drovandi et al., 2022). Due to joint pain and swelling of their wrists and ankles, patients are often misdiagnosed with juvenile idiopathic arthritis (JIA). There are anecdotal reports of improved joint symptoms in patients with MCTO following treatment with anti-rheumatic therapies (Mehawej et al., 2013; Upadia et al., 2018). However, objective evidence of improved inflammation has not been well-documented. In addition, anti-inflammatory medications are frequently discontinued following a genetic diagnosis of MCTO, preventing the ability to examine and further characterize the potential inflammatory aspects of MCTO.

MafB has been shown to negatively regulate receptor activator of nuclear factor kappaβ ligand (RANKL)-mediated osteoclastogenesis (Kim et al., 2007) and osteolysis has been regarded as the primary mechanism underlying the skeletal phenotype in MCTO. However, antiresorptive agents, such as bisphosphonates, have not proven effective at altering the trajectory of the disease and the carpal and tarsal bone destruction progresses over time. There is limited documentation in the published literature regarding the efficacy of denosumab, a RANKL inhibitor, in preventing or slowing down the progressive destruction of the affected periarticular bones in MCTO (Regev et al., 2021; Lerman et al., n.d.). These clinical observations suggest that the pathogenesis of MCTO may involve additional pathways than just osteoclast-mediated bone destruction (Ma et al., 2023).

The case-study presented herein demonstrates a relationship between joint symptoms and inflammation on imaging studies with longitudinal follow-up over 3–1/2 years. In addition, radiographs from an early age support the notion that abnormal bone formation may contribute to the skeletal phenotype of MCTO (Lazarus et al., 2017).

## 2 Case

**Fig. 1:**
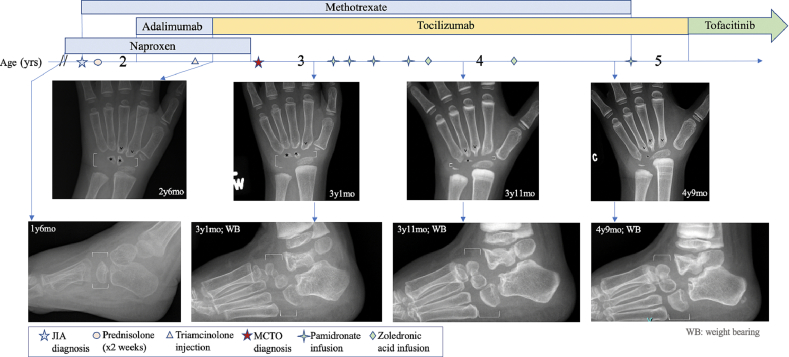
Left wrist and right ankle films from age 1 year 6 months to 4 years 9 months. Radiographs of the patient's wrist and ankle in relation to treatments received show progressive osteolysis and atypical bone formation. The joint compartments (represented by brackets) are narrow and hypoplastic with significantly decreased volume within the joint space. There are fewer carpal bones than expected for age, and the ones that are present (asterisks) appear qualitatively smaller and dystrophic from an early age. Increased erosions of the proximal metacarpals (v) are observed during treatment. The weight bearing bones of the mid- and hind-foot appear stable to slightly increased in size after initiating bisphosphonate treatment.

**Fig. 2:**
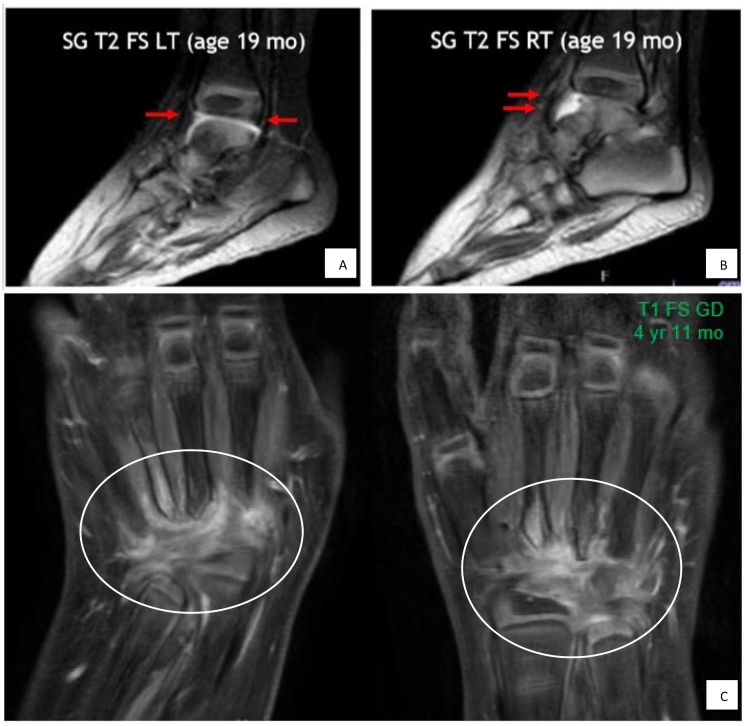
Joint inflammation on MRI. A, B: Presence of joint effusions and synovitis in bilateral ankle compartments at age 19 months. These images are prior to initiation of treatment with anti-rheumatic agents. C: Redemonstration of diffuse inflammation in bilateral wrists after discontinuation of methotrexate therapy.

A 15-month-old child (Ma, 2020) was evaluated for not standing or walking and crying when she placed weight on her feet and ankles. By age 18 months, she was ambulating on her knees. Her examination was pertinent for bilateral wrist and ankle swelling, stiffness, and decreased range of motion, tapered fingers, bilateral medial metatarsal curvatures, and minor dysmorphic features (shortened palpebral fissures, periorbital fullness, hypoplastic nares, underdeveloped philtrum, thin upper lip). Radiographs showed small wrist and ankle compartments and atypical size and shape of the carpal and tarsal bones (Fig. 1). A skeletal survey demonstrated no additional abnormalities in the remainder of the skeleton. Ankle MRI showed joint effusions and synovitis (Fig. 2A, B). She was initially diagnosed with JIA at age 21 months, and then later diagnosed with MCTO at age 33 months after whole exome sequencing identified a heterozygous *de novo* variant in *MAFB* (c.212C > G, p.Pro71Arg).

### 2.1 Inflammation

After she received a diagnosis of JIA, the patient began anti-rheumatic therapy to address her joint symptoms and the inflammation visible on imaging studies. She trialed various anti-rheumatic agents, including nonsteroidal anti-inflammatory drugs, methotrexate (MTX), adalimumab (tumor necrosis factor [TNF] inhibitor), and oral/intra-articular corticosteroids. The most effective treatment was MTX plus tocilizumab (IL-6 receptor antagonist), which led to pain relief and improved joint range of motion and overall function. After a few months of starting anti-rheumatic therapies, she began to weight bear and walked independently on her feet with orthotics by age 24 months. Her rheumatologist's clinical exam noted minimal active disease and musculoskeletal ultrasound showed no arthritis or tenosynovitis. Due to her considerable clinical improvement, and good tolerance of the medications without side effects, anti-rheumatic therapy was continued (Ma, 2020).

Later, MTX was discontinued at age 4 years 10 months due to frequent episodes of emesis that were felt to be a delayed idiosyncratic reaction to MTX. Within a few weeks of discontinuing MTX, she developed daily symptoms of painful wrists, hands, and fingers. These were not complaints that she had while taking MTX. Bilateral wrist MRI revealed diffuse inflammation of the wrists, extending to involve the proximal metacarpals (Fig. 2C).

The redemonstration of arthritic symptomatology in the context of diffuse inflammatory arthritis on MRI prompted treatment initiation with a different disease-modifying anti-rheumatic drug (DMARD), tofacitinib (pan-Janus kinase [JAK] inhibitor), at age 5 years 2 months. Tofacitinib is an approved drug for children with polyarticular JIA. It has been shown to repair bone erosions in patients with rheumatoid arthritis (Adam et al., 2020). There is also evidence that MafB is a downstream target of the JAK1/STAT3 signaling pathway (Gemelli et al., 2014).

Soon after starting tofacitinib, the joint symptoms improved and there was a burst of increased function and independence. The child was more active and able to do new activities, such as riding a scooter, climbing up a slide, and going up and down stairs without assistance. Her rheumatologist's exam noted no joint pain, swelling, warmth, or restricted range of motion.

After a few months of initiating tofacitinib therapy, the dose was increased due to a plateau in clinical improvement and improved but still apparent tenosynovitis and inflammatory changes on bilateral wrist MRI.

### 2.2 Dysfunctional bone formation

Radiographs since age 18 months revealed hypoplastic joint compartments. The joint space progressively narrowed even more over time (Fig. 1). In addition, the child persistently had fewer than the expected number of carpal bones for age (Greulich and Pyle, 1959). At age 2 years 6 months, 3 years, 3 years 6 months, 4 years, and 5 years, the patient had 67 %, 50 %, 40 %, 14 %, and 14 % of the expected number of carpal bones present in children without MCTO (Table 1). Of the carpal and tarsal bones that did form, they were visibly smaller and abnormally shaped compared to age-matched standards from a very young age.

**Table 1:** Persistently low number of carpal bones in a child with MCTO. From age 2 years 6 months to 5 years, the percentage of expected carpal bones in the patient's wrist was consistently lower compared to age-matched standards. Also, the number of carpal bones progressively declined, compared to increasing numbers over time in age-matched standards for children without MCTO.

Age	2 yr 6 mo	3 yr	3 yr 6 mo	4 yr	5 yr
Expected number of carpal bones without MCTO	3	4	5	7	7
Number of carpal bones in a child with MCTO	2^a^, ^b^	2^a^, ^b^	2^a^, ^b^	1^a^, ^b^	1^a^, ^b^
Percent of expected	67	50	40	14	14

a Carpal bones are also small and irregularly shaped.

b Wrist compartment and volume surrounding the carpal bones remain small and gets narrower with age.

Between ages 2 years 6 months and 3 years 1 month, interval osseous changes suggestive of osteolysis were observed in the wrists and ankles. Intravenous bisphosphonate therapy was initiated at that time using a cyclical pamidronate protocol not previously reported in patients with MCTO. The initial dose of pamidronate was 0.5 mg/kg and subsequent doses were 1 mg/kg at every 6–8-week intervals, not to exceed 9 mg/kg/year. After four pamidronate infusions, the patient switched to zoledronic acid 0.025 mg/kg every six months to reduce the frequency of infusions. During bisphosphonate treatment, MTX and tocilizumab were continued because of the clinical improvement in joint pain symptoms and inflammation.

While each class of drug as monotherapy had not proven effective in past reports, the simultaneous use of DMARDs with antiresorptive therapy had not been frequently reported in patients with MCTO. Serial radiographs of the wrists and ankles were obtained to examine the effect of combination therapy (DMARDs and bisphosphonates) on the bones of the wrists and ankles (Fig. 1). Unfortunately, the osteolysis in the wrists continued. The small, dystrophic carpal bones that were present became even smaller, or disappeared. By age 4 years 9 months, she had only one small carpal bone left. Also, the wrist joint space was markedly narrowed, and erosions of the proximal metacarpals were evident. Interestingly, the weight bearing bones of the mid- and hindfoot appeared to remain stable to slightly increased in size after initiating bisphosphonate treatment.

**Fig. 3:**
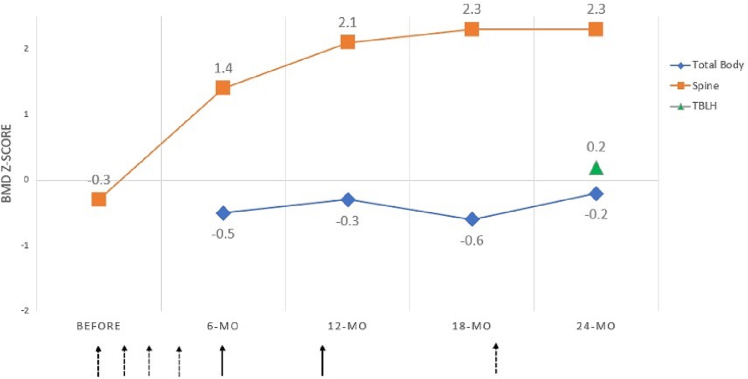
DXA data on bisphosphonate treatment. Spine BMD increased significantly from baseline on bisphosphonate treatment. Total body BMD remained stable in the normal range. Dashed arrows represent pamidronate infusions and the solid arrows represent zoledronic acid infusions. The patient received a total of five pamidronate and two zoledronic acid infusions over two years.

Bone turnover markers and bone mineral density (BMD) ascertained by dual-energy X-ray absorptiometry (DXA) were monitored while receiving bisphosphonate treatment (Fig. 3). Prior to the initial dose of pamidronate, bone-specific alkaline phosphatase was elevated (310.1 IU/L; 43.5–208.1) and procollagen 1 N-terminal propeptide (P1NP) was slightly greater than the upper limit of an age-dependent reference range (980 μg/L; 300–950) (Chubb et al., 2023). Serum c-telopeptide (CTX) was normal for age (824 pg/mL; 347–1508). P1NP and CTX increased six weeks after the initial pamidronate infusion to 1040 μg/L and 946 pg/mL, respectively. This may be due to having received just 50 % of the standard pamidronate dose as is customary for the initial infusion. All bone turnover markers significantly declined after completing 6–8 months of bisphosphonate treatment; bone-specific alkaline phosphatase was 205.2 IU/L, P1NP 385 μg/L, and CTX 667 pg/mL. After completing one year of zoledronic acid at six-month intervals, the P1NP and CTX trended upward to 660 μg/L and 906 pg/mL but remained within normal limits for age. Osteocalcin was checked once (19 days after the initial pamidronate infusion), and it was normal (96.3 ng/mL; 44–13).

The baseline bone mineral density (BMD) was normal for age and gender-matched peers with lumbar spine BMD Z-score −0.3 at age 3 years 2 months. The spine BMD increased significantly to +2.3 SD after two years of bisphosphonate treatment. The initial total body BMD was obtained after she received four pamidronate infusions. The total body BMD *Z*-score was normal at −0.5 SD and remained stable to slightly increased to −0.2 SD after two years of total bisphosphonate treatment. The total body-less head (TBLH) BMD Z-score was only available after she completed two years of bisphosphonate treatment. The TBLH BMD Z-score was normal at +0.2 SD at age 5 years 3 months and categorically similar to the TB skeletal site.

## 3 Discussion

MCTO is a rare skeletal dysplasia that presents as progressive osteolysis of the carpal and tarsal bones, nephropathy, and minor craniofacial differences. *MAFB* is the causative gene and mutations that cause MCTO cluster within a very narrow region of the transactivation domain (Zankl et al., 2012). Despite genetic homogeneity, there is significant phenotypic heterogeneity observed in patients with MCTO and there is not a clear genotype-phenotype correlation.

It is not uncommon for patients with MCTO to be misdiagnosed with JIA due to overlapping clinical symptoms and the higher prevalence of JIA compared to MCTO. Although many patients are initially treated with DMARDs, these agents are frequently discontinued after receiving a genetic diagnosis of MCTO due to perceived clinical ineffectiveness and inability to prevent joint disease progression. Therefore, the role of anti-rheumatic agents in the management of patients with MCTO is difficult to evaluate systematically with the available published reports.

That said, it is intriguing that patients with MCTO frequently present with joint pain, swelling, and decreased range of motion, which are symptoms of arthritis, or joint inflammation. Also, there are reports of imaging-proven inflammation and articular pain relief with TNF and IL-6 receptor inhibitors in patients with MCTO (Upadia et al., 2018; Nishikomori et al., 2015). One patient was described to have had several unsuccessful attempts to decrease etanercept (TNF inhibitor) therapy due to recurrent pain (Mehawej et al., 2013).

Our case expands the available literature of patients with MCTO and imaging-substantiated inflammation. Our patient experienced clinical symptoms of arthritis that correlated with clinical exam findings and inflammation on imaging studies on more than one occasion. Also, the timing of the recurrent joint pain soon after discontinuing MTX followed by improvement of pain and joint function after starting tofacitinib suggest that her anti-rheumatic therapies were controlling painful joint symptoms by managing joint inflammation.

Our patient remains ambulatory with the help of supportive braces and splints for her ankles and wrists. She also receives intensive physical and occupational therapy. It is possible that adequate pain management has been an under-recognized yet crucial part of her care for her to fully engage and continue these important therapies that strengthen her joints and improve function and mobility. Soon after starting tofacitinib, she was able to achieve new milestones such as riding a scooter, climbing up a slide, and going up and down stairs without assistance.

It remains unknown whether joint inflammation is chronic-persistent or presents intermittently and sporadically. It is also unclear if the inflammation may be occurring in response to osteolysis or as a guiding mechanism behind the pathogenesis of this condition. Nevertheless, it is important to better understand the role of inflammation in MCTO since inflammation, of any kind, is well-known to be detrimental to bone health. Meanwhile, it is plausible that inflammatory arthropathy may be a prevalent clinical feature of MCTO and DMARDs may play a role in the management of joint pain in a subset of patients with MCTO.

Osteolysis has been regarded as the primary mechanism underlying the skeletal phenotype of MCTO. However, potent antiresorptive treatments have been ineffective at preventing the progression of localized osteolysis in patients with MCTO. The cyclical pamidronate protocol that was used in our case delivered more frequent doses of bisphosphonate therapy in an effort to “trap-in” and preserve the ossification that had already occurred in the wrist and ankle joint spaces and prevent further osteolysis. The every 6–8-week pamidronate dosing protocol and its combination with DMARD therapies had not previously been tried in patients with MCTO. Unfortunately, this specific combination therapy did not significantly alter the clinical course of the wrists and progressive osteolysis of the carpal and proximal metacarpal bones was observed. On the other hand, the tarsal bones remained stable to slightly increase in size suggesting there may be a differential effect of this treatment combination on the weight bearing bones. Longer term follow-up is needed to determine whether these initial observations in the ankles persist. Also, more research is needed to determine if concurrent DMARDs and bisphosphonate (or denosumab) therapy can slow down or influence the pace of disease progression, which, if true, may prolong the duration of time that a child maintains some amount of joint function and mobility.

The idea that abnormal bone formation through the involvement of cartilage-producing chondrocytes may be contributing to the skeletal phenotype of MCTO has been presented in a few preclinical studies. In one study (Zhang and Ross, 2013), MafB was expressed in growth plate chondrocytes and MafB localized to the proliferative and hypertrophic zones of the growth plate cartilage in the neonatal rat femur. MafB was also shown to play a role in the formation of the cartilage matrix during chondrocyte differentiation through the regulation of aggrecan, matrix metalloproteinase (MMP)-3, and MMP-13 expression. Furthermore, in a healthy murine model, Lazarus and colleagues (Lazarus et al., 2017) hypothesized that MafB, along with MMP-2 and MMP-14, played an integral role in carpal and tarsal bone formation, having noted that MCTO, like other carpotarsal osteolysis disorders (such as multicentric osteolysis, nodulosis, and arthropathy or Torg [OMIM #259600] and Winchester [OMIM #277950] syndrome), had a specific skeletal site distribution. They demonstrated that MafB was expressed in carpal chondrocytes beginning at a young age of bone development and that MafB stained positive along the peripheral rim of mature bones in the murine forepaw, suggesting that abnormal periarticular bone modeling may be a primary mechanism underlying the skeletal phenotype of MCTO.

Our patient's case-study, to our knowledge, provides the first demonstration of dysfunctional bone formation as a contribution to the skeletal phenotype of a young child with MCTO and was a focus of the clinical report. It was possible to document the early history and progression of our patient's wrist and ankle bone involvement due to a genetic diagnosis of MCTO from a very young age. Her carpal and tarsal bones were visibly smaller and abnormal in shape compared to age-matched wrists and ankles without MCTO since age 18 months. She never formed all of the carpal bones and over time the percentage of carpal bones consistently declined such that she had only 14 % of the typical number of carpal bones by age five years. Additionally, the patient had hypoplastic wrist and ankle joint spaces since age 18 months that progressively narrowed over time, suggesting that the cartilaginous template may have been ill prepared to allow for the formation of typical carpal and tarsal bones. Taken together, our case supports the notion that abnormal bone formation, likely in combination with osteolysis, may play a role in the skeletal phenotype of MCTO.

BMD that falls within two standard deviations of the mean for age and gender-matched healthy controls is considered normal. Thus, our patient's spine BMD (−0.3 SD) was normal even prior to the initiation of bisphosphonate treatment. BMD data have been reported in other cases of MCTO and *Z*-scores range −0.3 to −1.4 at the spine and −1.1 at the hip (Mehawej et al., 2013; Ma, 2020; Chen et al., 2022). In a single case report where volumetric (v) BMD was ascertained by high-resolution peripheral quantitative computed tomography (HRpQCT), the total, trabecular, and cortical vBMD in the distal radius and total and cortical vBMD in the distal tibia were noted to be <3rd percentile of age-matched healthy volunteers (Regev et al., 2021). These data suggest that the osteopenia in MCTO may be region-specific and primarily affecting the peripheral (more so than axial) skeleton, which is where the commonly prototypical affected wrist and ankle bones are located. It is also possible that DXA grossly lacks the specificity that HRpQCT offers and is required to understand the bone characteristics and microarchitecture in MCTO. In addition, it has been suggested that there may be distinct pathogenic mechanisms that are responsible for the localized bone loss in the peripheral skeleton and unrelated to the systemic bone loss or generalized osteopenia that has been reported in MCTO (Regev et al., 2021; Lerman et al., n.d.).

We monitored our patient's response to bisphosphonate treatment with bone turnover markers and DXA BMD assessments. Our patient's serum CTX, a marker of bone resorption, was not elevated, but her markers of bone formation (bone-specific alkaline phosphatase and P1NP) were greater than the upper limit of an age-specific reference range. The significance of the increased bone formation markers without concurrent increase in serum CTX is uncertain, although osteoblasts are a source of RANKL and elevated levels of RANKL were reported by Regev and colleagues (Regev et al., 2021). Also, while her serum CTX was not overtly elevated, it may be relatively high for our specific patient and it decreased as expected with long-term bisphosphonate treatment.

Interestingly, the spine BMD increased more significantly than the total body BMD. A possible explanation is a higher rate of bone turnover in trabecular compared to cortical bone in our patient. In the case-study that reported HRpQCT data in a patient with MCTO, the trabecular number was reduced, and trabecular separation increased in the distal radius suggesting the trabecular bone microarchitecture may be affected in MCTO (Regev et al., 2021). Our patient's spine BMD was normal at baseline, suggesting that increased bone turnover was not clinically apparent, although perhaps it was low for her and improved with treatment. More studies are needed to better understand the role of bone turnover markers and bone properties of the general skeleton in MCTO. Meanwhile, it is sensible to monitor spine BMD and ideally additional skeletal sites until more information is gathered in the event there are differential effects of bisphosphonate treatment in patients with MCTO and to ensure there isn't overtreatment with bisphosphonate therapy.

In conclusion, MCTO is a rare skeletal dysplasia that frequently presents with joint pain and swelling and has a proclivity for the small carpal and tarsal bones of the wrists and ankles. There is very limited understanding of the specific molecular pathways underpinning the skeletal phenotype of MCTO. We presented a young child with MCTO whose joint symptoms correlated with inflammation on imaging studies more than once and demonstrated clinical improvement of joint symptoms following treatment with anti-rheumatic therapies. Joint inflammation may be a prevalent understudied aspect of MCTO and anti-rheumatic therapies may play a role in the acute management of joint pain in some patients. In addition, the early documentation of wrist and ankle radiographs in our patient demonstrated that her carpal and tarsal bones were abnormal from a very young age or failed to develop altogether. This observation suggests a component of dysfunctional chondrocytes or bone-forming cells precluding normal ossification of the carpal and tarsal bones in MCTO and why antiresorptive agents have offered limited therapeutic benefit at preventing bone disease progression. Generalizations cannot be made from a single case-study and more research is desperately needed to inform the use of safe and effective treatments in patients with MCTO.

## CRediT authorship contribution statement

**Bailey Trinkino:** Writing – review & editing, Writing – original draft, Data curation. **Nina S. Ma:** Writing – review & editing, Writing – original draft, Supervision, Methodology, Investigation, Funding acquisition, Formal analysis, Data curation, Conceptualization.

## Declaration of competing interest

BT: None.

NSM: NSM is an investigator for clinical trials that have been funded by Amgen, Ascendis Pharma, Calcilytix, Ultragenyx. NSM receives reviewer royalties from UpToDate. NSM is a scientific advisory board member of Sophie's Neighborhood.

## Data Availability

Data will be made available on request.
